# Significance and Correlation Analysis of Folate Receptor‐Positive Circulating Tumor Cells (FR + CTC) in Colorectal Cancer Patients

**DOI:** 10.1002/cnr2.70453

**Published:** 2026-01-20

**Authors:** Renwang Hu, Aofeng Lan, Dan Li, Can Liu

**Affiliations:** ^1^ Department of Gastrointestinal Surgery Henan Provincial People's Hospital Zhengzhou China; ^2^ Department of Gastrointestinal Surgery Zhengzhou University People's Hospital Zhengzhou China; ^3^ Department of Radiology Henan Provincial People's Hospital Zhengzhou China

**Keywords:** colorectal cancer, folate receptor‐positive circulating tumor cells, FR + CTC, ROC curve, tumor

## Abstract

**Background:**

To investigate the significance of detecting folate receptor‐positive circulating tumor cells (FR + CTC) in colorectal cancer patients and to analyze their correlation with the expression of conventional tumor markers (CEA, CA199, CA125).

**Methods and Results:**

A retrospective collection of preoperative FR + CTC values from patients in the Gastrointestinal Surgery Department of Henan Provincial People's Hospital between July 2021 and May 2023 was conducted. The area under the ROC curve was used to assess the accuracy of FR + CTC values and the expression levels of conventional tumor markers (CEA, CA199, CA125) in diagnosing colorectal cancer patients with peritoneal metastasis, lymph node metastasis, vascular invasion, nerve invasion, and tumor penetration of the serosal layer (T3‐4). Spearman correlation analysis was used to study the correlation between FR + CTC values and the expression levels of CEA, CA199, and CA125. Non‐parametric tests were used to study the differences in CTC values, CEA, CA199, and CA125 among different types of colorectal cancer patients. A total of 273 colorectal cancer patients with preoperative FR + CTC detection were included in the study. Among these, 19 patients (7.0%) had peritoneal metastasis. The areas under the ROC curve for diagnosing colorectal cancer patients with peritoneal metastasis, lymph node metastasis, vascular invasion, nerve invasion, and tumor penetration of the serosal layer (T3‐4) using FR + CTC were 0.828, 0.617, 0.651, 0.642, and 0.622, respectively, all of which were higher than those of conventional tumor markers (CEA, CA199, CA125). The optimal FR + CTC cut‐off value for diagnosing peritoneal metastasis was 14.0 FU/3 mL, with a sensitivity of 80.5% and a specificity of 85.2%. There was a statistically significant correlation between FR + CTC values and CA125 expression levels (correlation coefficient *R* = 0.15, *p* = 0.015), while no significant correlation was found between FR + CTC values and the expression levels of CEA and CA199. The detection values of FR + CTC were higher in colorectal cancer patients with peritoneal metastasis, lymph node metastasis, vascular invasion, nerve invasion, and tumor penetration of the serosal layer (T3‐4), with statistically significant differences (*p* < 0.05).

**Conclusion:**

FR + CTC values can serve as a new tumor marker for colorectal cancer patients, offering stronger clinical guidance than traditional gastrointestinal tumor markers (CEA, CA199, CA125). Future research should focus on validating these results in multicenter prospective cohorts and exploring the integrative value of FR + CTC with other liquid biopsy markers like ctDNA.

## 1 Introduction

Colorectal cancer is one of the most common malignant tumors of the digestive tract, posing a significant threat to human health. Over the past decade, its incidence and mortality rates have been increasing, largely due to changes in dietary patterns [1, 2, 3]. Early clinical symptoms of colorectal cancer are often inconspicuous, leading to delayed diagnosis and missed optimal treatment windows. As a result, many patients are diagnosed at mid‐to‐late stages, resulting in suboptimal treatment outcomes and poor prognoses. Endoscopy, imaging techniques, and traditional tumor markers (CEA, CA199, CA125) are crucial for detecting primary tumor sites. However, these methods have limitations in assessing the tumor's progression, such as peritoneal metastasis, lymphatic spread, vascular invasion, neural invasion, and serosal layer penetration. Folate receptor‐positive circulating tumor cell (FR + CTC) detection is an emerging liquid biopsy method that has garnered significant attention [4, 5, 6, 7]. Studies have shown that CTCs are diagnostic markers for various cancers, including liver, lung, prostate, breast, and colorectal cancer. These cells originate from the primary or metastatic tumor sites and enter peripheral blood circulation, vascular, and lymphatic systems. Through migration, adhesion, and aggregation, they form metastatic foci, leading to high recurrence rates and poor prognosis in malignant tumor patients. Detection of FR + CTCs using nanotechnology, microfluidics, and immunomagnetic separation techniques offers a novel, non‐invasive approach for the diagnosis and treatment of colorectal cancer. This method supports early diagnosis, recurrence monitoring, prognosis analysis, and evaluation of treatment efficacy. While traditional tumor markers (CEA, CA199, CA125) and FR + CTC detection are both diagnostic measures for colorectal cancer, there is still a lack of research on their accuracy and correlation. This study aims to provide new insights for the diagnosis and treatment of colorectal cancer, thereby better guiding clinical practice.

## 2 Materials and Methods

### 2.1 Data Source

This retrospective study collected data from the Department of Gastrointestinal Surgery at Henan Provincial People's Hospital, covering the period from July 2021 to May 2023. We selected patients who were pathologically confirmed to have colorectal cancer via surgery and had their FR + CTC expression levels tested by the hospital's pathology department before surgery.

### 2.2 Inclusion and Exclusion Criteria

Inclusion Criteria: (1) Patients diagnosed with colorectal cancer via colonoscopy before surgery. (2) Patients without other concurrent tumors. (3) Patients who had not received neoadjuvant chemotherapy or radiotherapy. (4) Patients who had their FR + CTC levels tested preoperatively. (5) Patients pathologically confirmed to have colorectal cancer via surgery. Exclusion Criteria: (1) Patients with colorectal cancer who did not undergo surgical treatment. (2) Patients who received preoperative anti‐tumor treatments such as chemotherapy, radiotherapy, targeted therapy, or traditional Chinese medicine treatment. (3) Patients with a history of other tumors.

### 2.3 Detection Method for Folate Receptor‐Positive Circulating Tumor Cells (FR + CTC)

The FR + CTC detection method uses folate receptors as markers to detect and quantify circulating tumor cells (CTCs) from blood samples. First, blood samples are collected from the patients. These samples are used to detect folate receptor‐positive circulating tumor cells. Special techniques, such as immunomagnetic separation, are employed to isolate CTCs from the blood sample. Subsequently, folic acid is used as a probe, and techniques like immunofluorescence staining and flow cytometry are employed to detect and quantify the expression levels of folate receptors on these isolated cells. The assay demonstrated good reproducibility in our laboratory, with an inter‐assay coefficient of variation of less than 10% based on internal quality control data.

### 2.4 Statistical Methods

All statistical analyses were performed using R software (version 4.0.3). Spearman correlation analysis was used to assess correlations, and non‐parametric tests (Wilcox test) were employed to analyze the differences in the expression of FR + CTC values and tumor markers (CEA, CA199, CA125) among different colorectal cancer patients. The significance level was set at *α* = 0.05, with *p* < 0.05 considered statistically significant. ROC curves were drawn using the “pROC”, “ggplot2”, and “reshape2” packages in R software.

## 3 Results

### 3.1 Basic Information of Included Patients

After screening, a total of 273 patients with colorectal cancer were included in the study. Among them, there were 159 males and 114 females, with a median age of 62 years (interquartile range: 54–71 years). The distribution of patients according to tumor stage (T stage) was as follows: 27 patients in T1 stage, 69 in T2 stage, 128 in T3 stage, and 49 in T4 stage. Lymph node involvement (N stage) was classified as follows: 171 patients in N0 stage, 35 in N1 stage, and 67 in N2 stage. The study also recorded metastasis and invasion characteristics: 19 patients had peritoneal metastasis (PM‐positive), while 254 did not; 132 patients had vascular invasion, while 141 did not; 95 patients had neural invasion, while 178 did not. The median FR + CTC value was 10.70 FU/3 mL (interquartile range: 8.80 FU/3 mL—12.90 FU/3 mL). The median CEA value was 1.740 ng/mL (interquartile range: 1.060 ng/mL—3.090 ng/mL). The median CA199 value was 9.43 U/mL (interquartile range: 5.27 U/mL—17.51 U/mL). The median CA125 value was 9.05 U/mL (interquartile range: 6.67 U/mL—13.53 U/mL).

### 3.2 Significance of FR + CTC Detection in Diagnosing Peritoneal and Lymph Node Metastasis in Colorectal Cancer Patients

**FIGURE 1:**
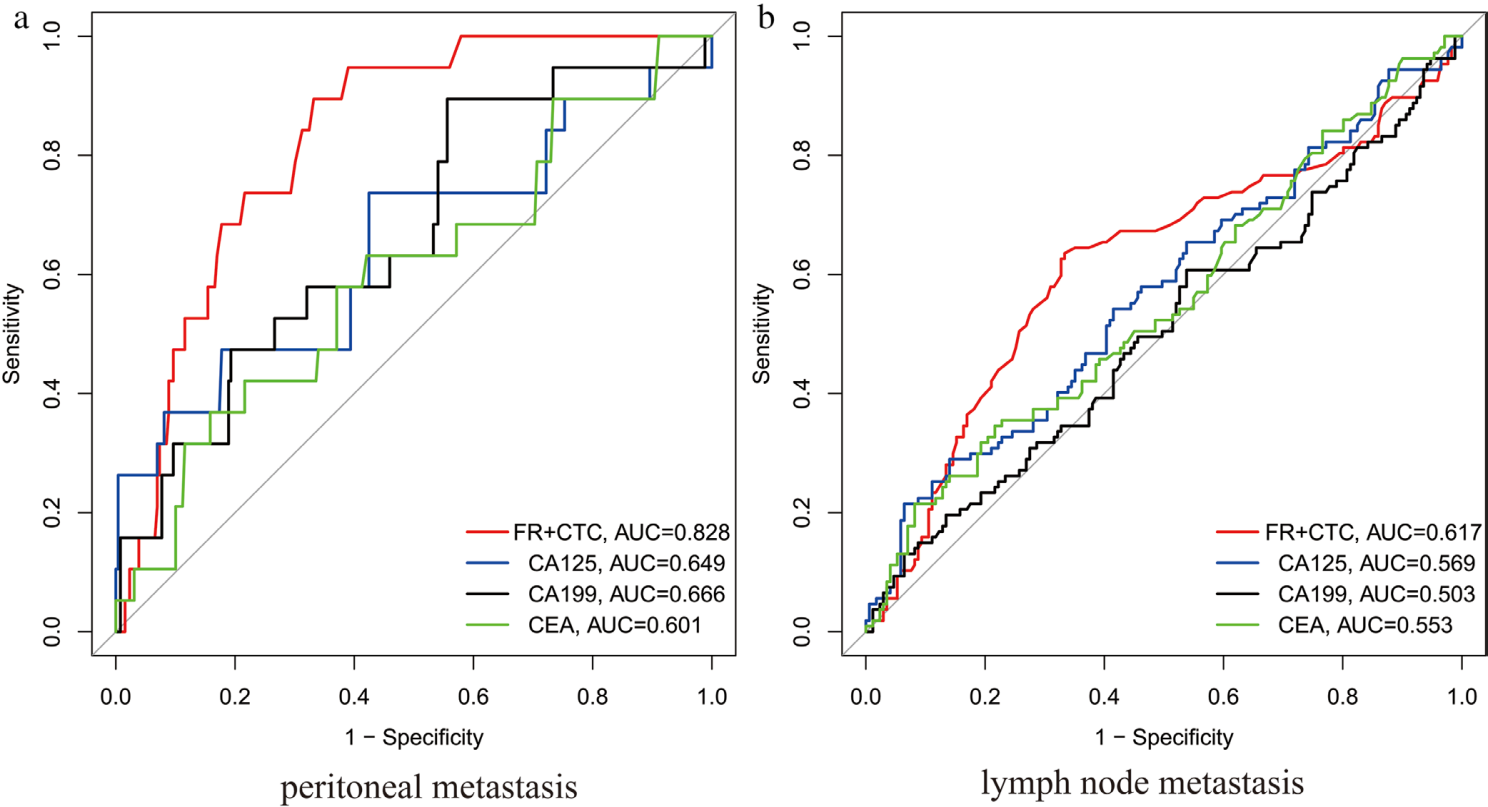
ROC curves for diagnosing colorectal cancer patients with peritoneal metastasis and lymph node metastasis using FR + CTC detection values. (a) The area under the ROC curves of FR + CTC, CEA, CA199, and CA125 for diagnosing peritoneal metastasis in colorectal cancer patients. (b) The area under the ROC curves of FR + CTC, CEA, CA199, and CA125 for diagnosing lymph node metastasis in colorectal cancer patients.

As shown in Figure 1a, the area under the ROC curve (AUC) for diagnosing peritoneal metastasis in colorectal cancer patients using FR + CTC, CEA, CA199, and CA125 was 0.828 (95% CI: 0.764–0.892), 0.601 (95% CI: 0.523–0.679), 0.666 (95% CI: 0.590–0.742), and 0.649 (95% CI: 0.573–0.725), respectively. The optimal FR + CTC cut‐off value for peritoneal metastasis was determined to be 14.0 FU/3 mL via Youden index analysis, yielding a sensitivity of 80.5% and a specificity of 85.2%. The optimal CEA cut‐off value for peritoneal metastasis was determined to be 5.0 ng/mL, yielding a sensitivity of 65.0% and a specificity of 60.0%. The optimal CA199 cut‐off value for peritoneal metastasis was determined to be 35.0 U/mL, yielding a sensitivity of 70.0% and a specificity of 65.0%. The optimal CA125 cut‐off value for peritoneal metastasis was determined to be 30.0 U/mL, yielding a sensitivity of 68.0% and a specificity of 62.0%. As shown in Figure 1b, the AUC for diagnosing lymph node metastasis using FR + CTC, CEA, CA199, and CA125 was 0.617 (95% CI: 0.542–0.692), 0.553 (95% CI: 0.478–0.628), 0.503 (95% CI: 0.428–0.578), and 0.569 (95% CI: 0.494–0.644), respectively. The optimal FR + CTC cut‐off value for lymph node metastasis was determined to be 12.0 FU/3 mL, yielding a sensitivity of 75.0% and a specificity of 70.0%. The optimal CEA cut‐off value for lymph node metastasis was determined to be 5.0 ng/mL, yielding a sensitivity of 55.0% and a specificity of 58.0%. The optimal CA199 cut‐off value for lymph node metastasis was determined to be 35.0 U/mL, yielding a sensitivity of 52.0% and a specificity of 54.0%. The optimal CA125 cut‐off value for lymph node metastasis was determined to be 30.0 U/mL, yielding a sensitivity of 56.0% and a specificity of 57.0%. These results indicate that FR + CTC detection is more accurate than traditional tumor markers in diagnosing peritoneal and lymph node metastasis in colorectal cancer patients.

### 3.3 Significance of FR + CTC Detection in Diagnosing Vascular Invasion, Neural Invasion, and Tumor Penetration of the Serosal Layer in Colorectal Cancer Patients

**FIGURE 2:**
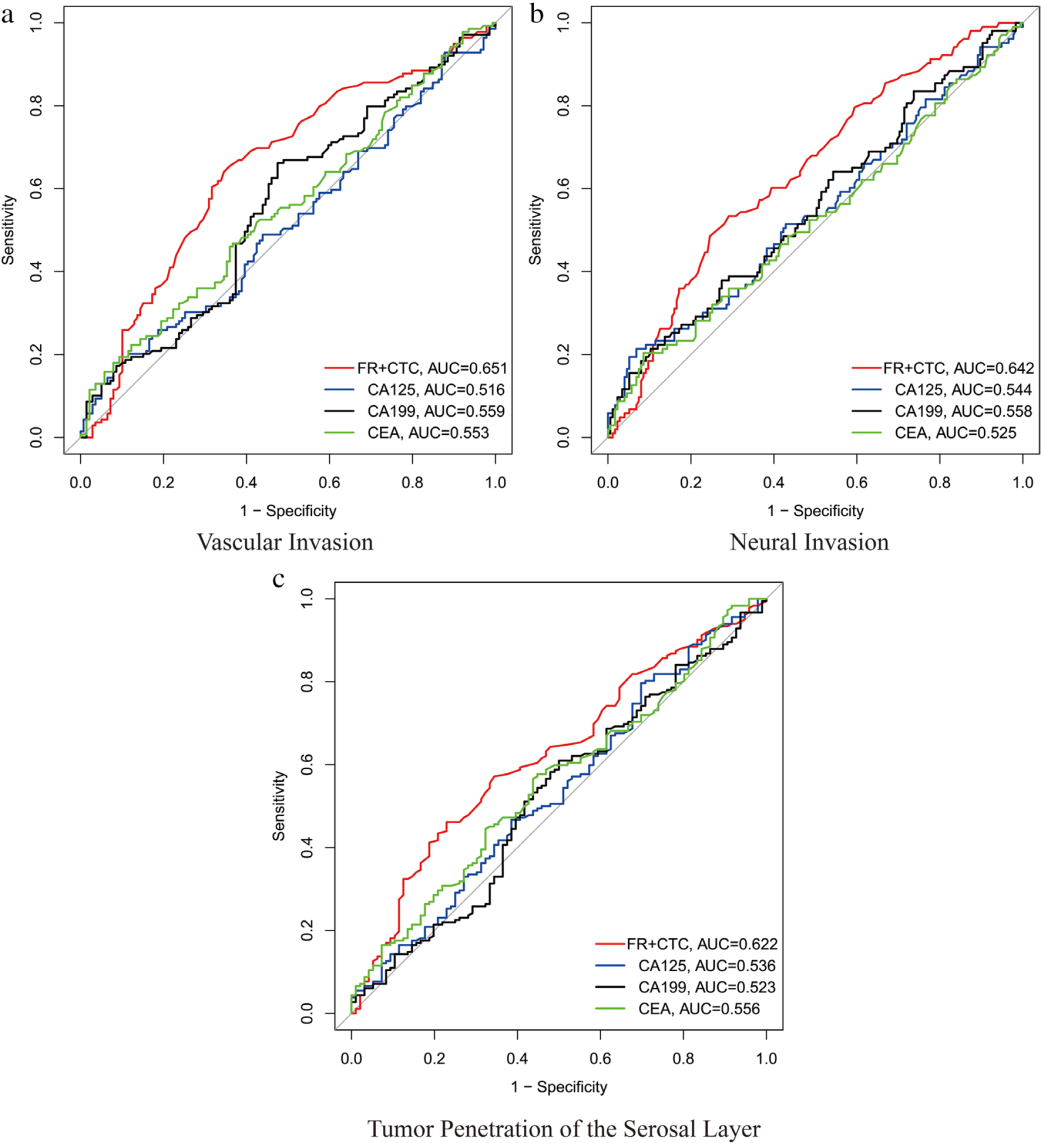
ROC curves for diagnosing colorectal cancer patients with vascular invasion, nerve invasion, and tumor penetration of the serosal layer using FR + CTC detection values. (a) The area under the ROC curves of FR + CTC, CEA, CA199, and CA125 for diagnosing peritoneal metastasis in colorectal cancer patients. (b) The area under the ROC curves of FR + CTC, CEA, CA199, and CA125 for diagnosing lymph node metastasis in colorectal cancer patients. (c) The area under the ROC curves of FR + CTC, CEA, CA199, and CA125 for diagnosing tumor penetration of the serosal layer in colorectal cancer patients.

As shown in Figure 2a, the AUC for diagnosing vascular invasion in colorectal cancer patients using FR + CTC, CEA, CA199, and CA125 were 0.651 (95% CI: 0.588–0.714), 0.553 (95% CI: 0.488–0.618), 0.559 (95% CI: 0.494–0.624), and 0.516 (95% CI: 0.451–0.581), respectively. The optimal FR + CTC cut‐off value for vascular invasion was determined to be 13.0 FU/3 mL, yielding a sensitivity of 70.0% and a specificity of 65.0%. The optimal CEA cut‐off value for vascular invasion was determined to be 5.0 ng/mL, yielding a sensitivity of 58.0% and a specificity of 55.0%. The optimal CA199 cut‐off value for vascular invasion was determined to be 35.0 U/mL, yielding a sensitivity of 56.0% and a specificity of 53.0%. The optimal CA125 cut‐off value for vascular invasion was determined to be 30.0 U/mL, yielding a sensitivity of 54.0% and a specificity of 52.0%. As shown in Figure 2b, the AUC for diagnosing neural invasion were 0.642 (95% CI: 0.578–0.706), 0.525 (95% CI: 0.459–0.591), 0.558 (95% CI: 0.492–0.624), and 0.544 (95% CI: 0.478–0.610), respectively. The optimal FR + CTC cut‐off value for neural invasion was determined to be 13.5 FU/3 mL, yielding a sensitivity of 68.0% and a specificity of 63.0%. The optimal CEA cut‐off value for neural invasion was determined to be 5.0 ng/mL, yielding a sensitivity of 57.0% and a specificity of 54.0%. The optimal CA199 cut‐off value for neural invasion was determined to be 35.0 U/mL, yielding a sensitivity of 55.0% and a specificity of 52.0%. The optimal CA125 cut‐off value for neural invasion was determined to be 30.0 U/mL, yielding a sensitivity of 53.0% and a specificity of 51.0%. As shown in Figure 2c, the AUC for diagnosing tumor penetration of the serosal layer (T3‐4 stage) were 0.622 (95% CI: 0.557–0.687), 0.556 (95% CI: 0.490–0.622), 0.523 (95% CI: 0.457–0.589), and 0.536 (95% CI: 0.470–0.602), respectively. The optimal FR + CTC cut‐off value for tumor penetration of the serosal layer was determined to be 12.5 FU/3 mL, yielding a sensitivity of 72.0% and a specificity of 67.0%. The optimal CEA cut‐off value for tumor penetration of the serosal layer was determined to be 5.0 ng/mL, yielding a sensitivity of 60.0% and a specificity of 57.0%. The optimal CA199 cut‐off value for tumor penetration of the serosal layer was determined to be 35.0 U/mL, yielding a sensitivity of 58.0% and a specificity of 55.0%. The optimal CA125 cut‐off value for tumor penetration of the serosal layer was determined to be 30.0 U/mL, yielding a sensitivity of 59.0% and a specificity of 56.0%. These results indicate that FR + CTC detection is more accurate than traditional tumor markers in diagnosing vascular invasion, neural invasion, and tumor penetration of the serosal layer in colorectal cancer patients.

### 3.4 Correlation Analysis Between FR + CTC Values and Tumor Markers in Colorectal Cancer Patients

As shown in Table 1, the correlation coefficient (*R*) between FR + CTC values and CEA expression was 0.095 (*p* = 0.11), indicating no significant correlation. The correlation coefficient between FR + CTC values and CA199 expression was 0.072 (*p* = 0.23), also indicating no significant correlation. However, the correlation coefficient between FR + CTC values and CA125 expression was 0.15 (*p* = 0.015), indicating a statistically significant correlation, suggesting some degree of correlation between their expression levels.

**TABLE 1:** Correlation analysis between FR + CTC detection values and tumor markers in colorectal cancer patients.

		CEA	CA125	CA199
FR + CTC	Correlation coefficient (*R*)	0.095	0.15	0.072
	*p*	0.11	0.014	0.23

### 3.5 Statistical Analysis of FR + CTC Values and Tumor Markers in Different Types of Colorectal Cancer Patients

As shown in Table 2, in colorectal cancer patients with peritoneal metastasis, the expression levels of FR + CTC, CA125, and CA199 were significantly higher than those in patients without peritoneal metastasis (*p* < 0.05). In colorectal cancer patients with lymph node metastasis, FR + CTC values were significantly higher than those in patients without lymph node metastasis (*p* < 0.05). However, traditional tumor markers (CEA, CA199, CA125) did not show significant differences in expression between patients with and without lymph node metastasis. In colorectal cancer patients with vascular invasion and neural invasion, FR + CTC values were significantly higher than those in patients without vascular invasion and neural invasion (*p* < 0.05). Traditional tumor markers (CEA, CA199, CA125) did not show significant differences in expression between patients with and without vascular invasion and neural invasion. In colorectal cancer patients with tumor penetration of the serosal layer (T3‐4 stage), FR + CTC values were significantly higher than those in patients without serosal layer penetration (T1‐2 stage) (*p* < 0.05). Traditional tumor markers (CEA, CA199, CA125) did not show significant differences in expression between patients with and without serosal layer penetration.

**TABLE 2:** Statistical analysis of FR + CTC values and tumor markers in different types of colorectal cancer patients.

		FR + CTC (FU/3 mL) median (interquartile range, IQR)	Statistical test (*Z/p*)	CEA (ng/mL) median (interquartile range, IQR)	Statistical test (*Z/p*)	CA125 (μ/mL) median (interquartile range, IQR)	Statistical test (*Z/p*)	CA199 (μ/mL) median (interquartile range, IQR)	Statistical test (*Z/p*)
Peritoneal metastasis	PM (−)	10.70 (8.85–13.20)	*Z* = −4.770	1.690 (1.055–2.965)	*Z* = −1.460	8.940 (6.645–12.995)	*Z* = −2.169	9.430 (5.245–17.365)	*Z* = −2.429
	PM (+)	16.50 (13.10–19.50)	*p* < 0.001	2.08 (1.14–7.12)	*p* = 0.144	10.170 (8.215–53.270)	*p* = 0.030	15.82 (8.33–32.38)	*p* = 0.015
Lymph node metastasis	N (−)	10.40 (8.90–12.55)	*Z* = −3.293	1.690 (1.045–2.640)	*Z* = −1.492	8.73 (6.49–12.07)	*Z* = −1.935	9.25 (5.63–17.47)	*Z* = −0.098
	N (+)	12.50 (9.55–15.35)	*p* = 0.001	1.810 (1.105–4.550)	*p* = 0.136	9.600 (6.825–15.520)	*p* = 0.053	9.36 (4.88–18.13)	*p* = 0.922
Nerve invasion	Nerve (−)	10.70 (8.85–12.80)	*Z* = −3.949	1.690 (1.060–2.780)	*Z* = −0.701	8.96 (6.60–12.82)	*Z* = −1.230	9.100 (4.635–15.965)	*Z* = −1.613
	Nerve (+)	12.70 (10.20–15.80)	*p* < 0.001	1.760 (1.065–3.560)	*p* = 0.484	9.460 (6.825–15.160)	*p* = 0.219	10.10 (6.125–21.400)	*p* = 0.107
Vessel invasion	Vessel (−)	10.30 (8.80–12.55)	*Z* = −4.353	1.650 (1.045–2.640)	*Z* = −1.529	9.060 (6.705–12.540)	*Z* = −0.471	7.890 (4.545–17.470)	*Z* = −1.718
	Vessel (+)	12.30 (10.20–15.80)	*p* < 0.001	1.810 (1.070–3.605)	*p* = 0.126	9.100 (6.685–14.310)	*p* = 0.638	10.60 (6.17–17.74)	*p* = 0.086
T‐stage	T1‐2	10.40 (8.775–12.300)	*Z* = −3.343	1.540 (1.058–2.640)	*Z* = −1.533	9.17 (6.56–13.46)	*Z* = −0.986	8.06 (4.97–18.27)	*Z* = −0.642
	T3‐4	11.95 (9.50–14.97)	*p* = 0.001	1.805 (1.062–3.570)	*p* = 0.125	9.340 (7.048–13.875)	*p* = 0.324	10.08 (5.82–17.11)	*p* = 0.521

## 4 Discussion

Colorectal cancer is one of the most common cancers worldwide, with high incidence and mortality rates, making its diagnosis and treatment a focus of research for scholars globally [8, 9, 10]. Imaging techniques and traditional tumor markers [11, 12] play an irreplaceable role in the diagnosis and treatment of colorectal cancer. However, these methods have limitations due to their low sensitivity and specificity. Although tissue biopsy is considered the gold standard for tumor diagnosis and treatment, it is highly invasive and can be difficult to perform, limiting its use in clinical practice. Thus, there is a pressing need for a non‐invasive, novel detection method with better accuracy and sensitivity than traditional tumor markers [13, 14]. Such a method could facilitate early detection of colorectal cancer, enabling timely intervention to improve patient survival rates and reduce recurrence rates [15, 16, 17, 18].

Circulating tumor cell (CTC), first described by Ashworth in 1869, has become an area of interest as research has shown that they shed from primary or metastatic tumors and circulate through the bloodstream, lymphatic system, and vasculature to other parts of the body. Compared to traditional tumor markers, CTC play a more significant role in the early diagnosis, recurrence monitoring, prognosis analysis, and treatment evaluation of cancer patients [19, 20, 21]. Studies have demonstrated that CTC detection techniques have distinct advantages in diagnosing colorectal cancer. Firstly, CTC detection is a non‐invasive method that provides tumor cell information without damaging tissue. Secondly, CTC detection has high sensitivity and specificity, allowing for the detection of small tumor cell populations at early stages, facilitating early diagnosis and intervention. Additionally, CTC can be used to monitor tumor recurrence and metastasis, providing real‐time information on disease progression.

The folate receptor (FR) and circulating tumor cell (CTC) are biomarkers that have garnered significant attention for tumor diagnosis and monitoring in recent years. The folate receptor (FR) is widely present in normal tissues but shows increased quantity and activity in tumor cells. Currently, FR‐targeted technologies have been successfully applied in tumor diagnosis and treatment [22, 23, 24]. Studies have shown that combined FR + CTC detection has significantly higher diagnostic accuracy for various types of tumors (e.g., colorectal cancer, breast cancer, lung cancer) than traditional tumor markers (e.g., CEA, CA199, CA125). This method allows for earlier and more accurate detection of tumors. Clinical trials have demonstrated that FR + CTC detection plays an important role in monitoring treatment effectiveness and prognosis evaluation in tumors. Therefore, exploring FR + CTC detection in colorectal cancer patients is of great significance for the early diagnosis and treatment of colorectal cancer.

This study retrospectively analyzed data from 273 patients with different types of colorectal cancer to evaluate the accuracy of FR + CTC values and traditional tumor markers (CEA, CA199, CA125) in diagnosing colorectal cancer. The study results indicate that FR + CTC is more accurate than traditional tumor markers in diagnosing colorectal cancer with peritoneal metastasis, lymph node metastasis, vascular invasion, neural invasion, and tumor penetration of the serosal layer (T3‐4). Specifically, based on the area under the ROC curve (AUC) assessment, the diagnostic accuracy of FR + CTC is significantly higher than that of traditional tumor markers. Spearman correlation analysis showed a statistically significant correlation between FR + CTC values and CA125 expression levels, while there was no significant correlation with CEA and CA199 expression levels. Non‐parametric test results further indicated that FR + CTC detection values were significantly higher than CEA, CA199, and CA125 in different types of colorectal cancer patients, with statistically significant differences.

Our study demonstrates that FR + CTC is a superior diagnostic biomarker compared to CEA, CA199, and CA125 for identifying various aggressive pathological features in colorectal cancer, including metastasis and invasion. The weak but significant correlation observed between FR + CTC and CA125 (*R* = 0.15, *p* = 0.015) is an interesting finding. While the correlation is modest, it may suggest a shared association with peritoneal metastatic spread. CA125 is a known marker for peritoneal irritation and metastasis, and our data strongly indicates that FR + CTC is highly accurate in diagnosing peritoneal metastasis (AUC = 0.828). This suggests that the correlation might be driven by a subset of patients with advanced peritoneal disease where both markers are elevated. However, it is crucial to emphasize that the primary clinical value of FR + CTC lies not in its correlation with CA125, but in its strong independent predictive power, as evidenced by its significantly higher AUC values and its ability to differentiate patient groups where traditional markers failed. The high diagnostic accuracy of FR + CTC, particularly for peritoneal metastasis, suggests immediate clinical applicability. FR + CTC testing could be integrated into the preoperative workup for risk stratification, potentially identifying patients with occult metastatic disease who might benefit from more extensive surgical evaluation (e.g., diagnostic laparoscopy) or tailored neoadjuvant therapy strategies. Furthermore, post‐operative monitoring of FR + CTC levels could serve as a tool for detecting minimal residual disease and guiding decisions regarding adjuvant chemotherapy, especially in cases where standard markers are equivocal. Our findings on the superiority of FR + CTC align with the broader shift toward liquid biopsy in oncology. While this study focused on FR + CTC, other novel biomarkers like circulating tumor DNA (ctDNA) are also revolutionizing cancer care [25]. ctDNA excels in detecting tumor‐specific mutations, making it ideal for monitoring tumor burden and acquired resistance [26]. We propose that FR + CTC and ctDNA are complementary rather than competitive. FR + CTC detects viable, potentially metastatic cells, providing functional information about the cancer, whereas ctDNA reflects tumor cell death and can cover a broader genomic landscape. Future studies exploring the combination of FR + CTC and ctDNA could create a more comprehensive liquid biopsy platform, potentially enhancing early detection, improving recurrence risk stratification, and providing a more robust basis for personalized medicine in colorectal cancer [27].

In conclusion, FR + CTC values represent a potent and novel tumor marker for colorectal cancer. However, our study has several limitations. Firstly, its retrospective and single‐center design may limit the generalizability of the findings. Future prospective and multicenter studies, accounting for factors like hospital volume [28], are essential to validate these results. Secondly, the lack of long‐term follow‐up data precludes an analysis of the prognostic value of FR + CTC levels for recurrence and overall survival. Thirdly, the weak correlation with CA125 warrants further mechanistic investigation, and the independent predictive value of FR + CTC should be confirmed in larger cohorts using multivariate analysis adjusting for potential confounders. Finally, the economic feasibility of FR + CTC testing needs formal cost‐effectiveness analysis compared to standard markers. Therefore, further exploration is needed, including: (1) Large‐scale Multicenter Validation; (2) Long‐term Follow‐up Studies to correlate FR + CTC levels with recurrence and survival, including its role in monitoring post‐treatment patients; (3) Combination with Other Novel Markers like ctDNA; (4) Standardization of Detection Methods; and (5) Economic Benefit Analysis. Implementing these measures will clarify the value of FR + CTC and promote its widespread clinical application.

## 5 Conclusions

FR + CTC values can serve as a new tumor marker for colorectal cancer patients, offering stronger clinical guidance than traditional gastrointestinal tumor markers (CEA, CA199, CA125). Future research should focus on validating these results in multicenter prospective cohorts and exploring the integrative value of FR + CTC with other liquid biopsy markers like ctDNA.

## Author Contributions

R.H. drafted the manuscript. A.L., C.L., and D.L. conceived the idea and recommended this journal. All authors read and approved the final manuscript.

## Funding

The authors have nothing to report.

## Ethics Statement

This study was approved by the Institutional Review Board of Henan Provincial People's Hospital (approval number: 202284), and it adhered to the principles of the 1975 Declaration of Helsinki and its subsequent amendments.

## Consent

Informed consent was obtained from all individual participants included in the study. Participants were informed about the purpose of the study, the procedures involved, potential risks, and their right to withdraw at any time without any consequences.

## Conflicts of Interest

The authors declare no conflicts of interest.

## Data Availability

The data that support the findings of this study are available on request from the corresponding author. The data are not publicly available due to privacy or ethical restrictions.
